# First person – Danny Legge

**DOI:** 10.1242/dmm.039412

**Published:** 2019-03-07

## Abstract

First Person is a series of interviews with the first authors of a selection of papers published in Disease Models & Mechanisms (DMM), helping early-career researchers promote themselves alongside their papers. Danny Legge is first author on ‘BCL-3 promotes a cancer stem cell phenotype by enhancing β-catenin signalling in colorectal tumour cells’, published in DMM. Danny conducted the research described in this article while a PhD student in Professor Ann Williams's lab at Colorectal Tumour Biology Group, School of Cellular and Molecular Medicine, University of Bristol, UK. He is now a postdoc in the lab of Dr Keith Brown at Cancer Epigenetics Laboratory, School of Cellular and Molecular Medicine, University of Bristol, UK, investigating the role of cancer stem cells in colorectal cancer.


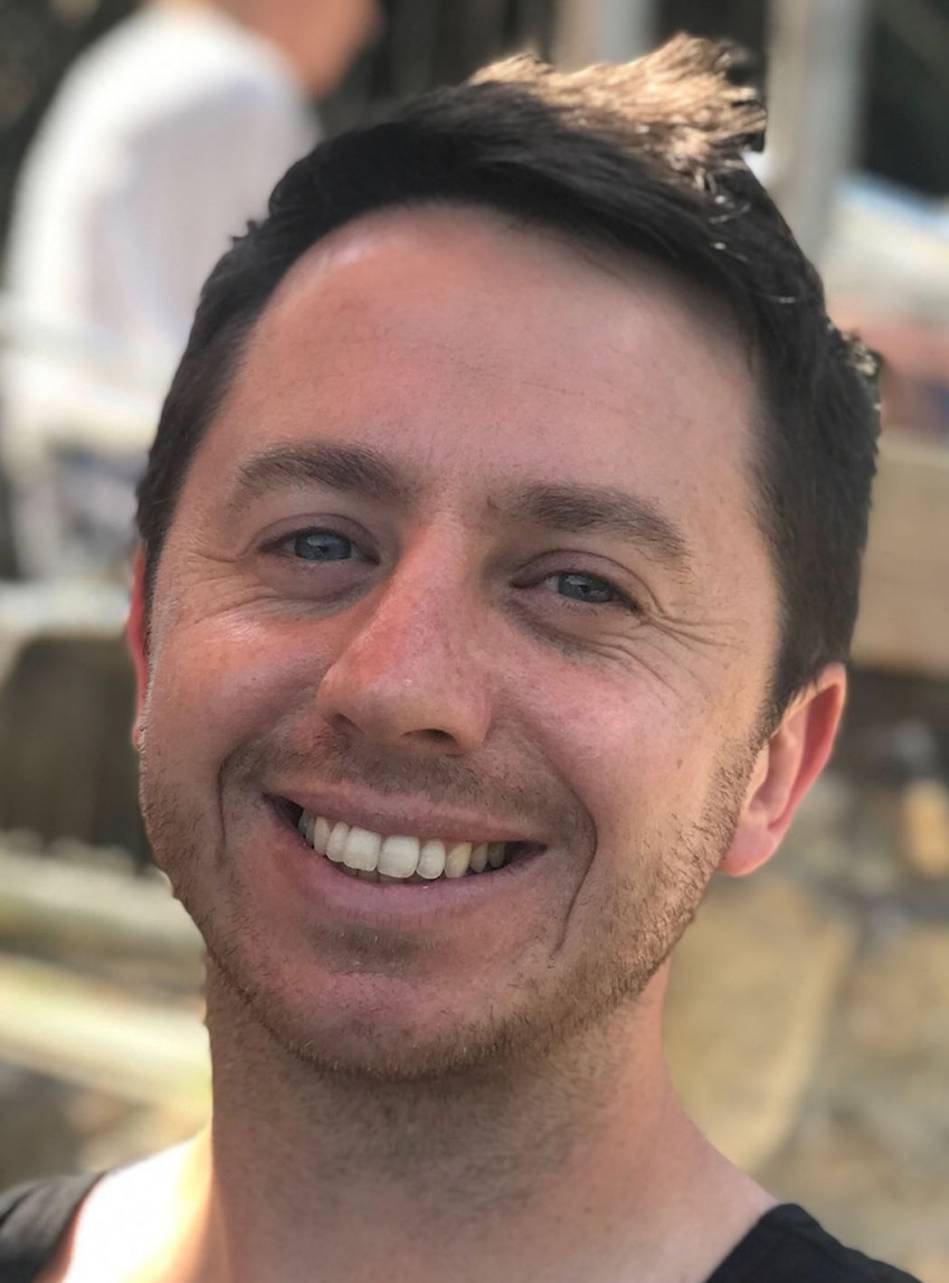


**Danny Legge**

**How would you explain the main findings of your paper to non-scientific family and friends?**

Colorectal cancer (bowel cancer) is the second most common cause of cancer mortality in the UK. It is thought that cancer stem cells are responsible for the initiation of colorectal tumours and fuel the regeneration of tumours following the end of treatment. It is hypothesized that destruction of the cancer stem cells is required to prevent relapse of the cancer. Mutation of normal intestinal stem cells or reversion of specialized cells (de-differentiation) to a more stem-like state is suggested to be the origin of cancer stem cells, with both normal stem cells and cancer stem cells sharing molecular signatures. Many of the genes in these signatures are targets of the Wnt signalling pathway and almost all colorectal cancers undergo deregulated Wnt signalling. The β-catenin protein is the main effector of this pathway. Our study shows that BCL-3, a protein induced by inflammation, is a novel regulator of Wnt/β-catenin signalling and highlights the importance of this interaction for cancer stem cell function. Our results indicate that this protein could be targeted to reduce de-differentiation/plasticity of cancer cells and therefore target the cancer stem cell phenotype, providing more effective therapies for colorectal cancer patients in the future.

**What are the potential implications of these results for your field of research?**

Plasticity of cancer stem cells is recognized as a significant barrier to effective treatments. The ability of cells to de-differentiate, as promoted by the microenvironment, needs to be overcome to prevent tumour regeneration. Targeting LGR5-positive cancer stem cells alone has been shown to be ineffective, as surrounding tumour cells de-differentiate to re-express LGR5 and refuel tumour growth. Our data show that BCL-3 promotes expression of the intestinal stem cell markers LGR5 and ASCL2 in colorectal cancer cells. By targeting BCL-3, it may be possible to reduce this de-differentiation capacity, in addition to suppressing levels of aberrant Wnt signalling, given that BCL-3 functions downstream of mutated APC and β-catenin (the two most frequent driver mutations in colorectal tumours) to modulate Wnt signalling.

**What are the main advantages and drawbacks of the model system you have used as it relates to the disease you are investigating?**

Three-dimensional organoid and spheroid culture are valuable *ex vivo* tools that allow us to study colorectal cancer in more physiologically relevant conditions than 2D cell culture work alone. They provide a stepping stone between cell lines and *in vivo* mouse models. Future work will involve the use of mouse models to understand the role of BCL-3 in cancer stem cell fate *in vivo.*

“Developing models that replicate the tumour microenvironment more closely will be key to unlocking the potential of targeting cancer stem cells in colorectal cancer.”

**What has surprised you the most while conducting your research?**

The BCL-3-mediated regulation of only a subset of Wnt targets involved in maintaining the intestinal stem cell phenotype is intriguing. Our work has shown that not all Wnt targets are regulated by BCL-3; in fact, many of the canonical proliferation-inducing targets usually associated with Wnt signalling are not regulated by modulating BCL-3 levels. Understanding how this is achieved may provide novel insights into the mechanisms of fine-tuning β-catenin/TCF-mediated transcription.

**Figure:**
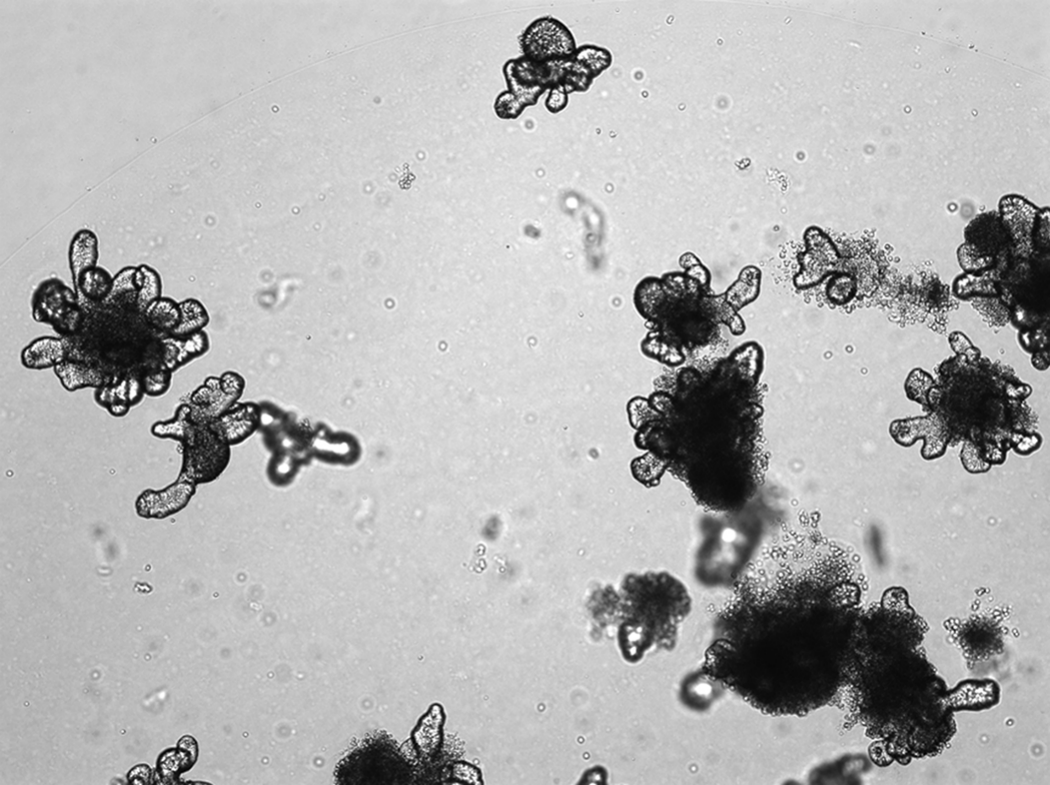
**Widefield image of intestinal organoids derived from mouse small intestine.**

**Describe what you think is the most significant challenge impacting your research at this time and how will this be addressed over the next 10 years?**

Developing models that replicate the tumour microenvironment more closely will be key to unlocking the potential of targeting cancer stem cells in colorectal cancer. The importance of targeting the plasticity of cancer stem cells has already been highlighted, with many of these factors, such as high levels of Wnt signalling and other growth factors in the microenvironment, shown to enhance LGR5 levels in previously differentiated cells. Co-culture of patient-derived tumour organoids with associated mesenchymal and/or immune cells may provide useful models to study the interactions and signalling between tumours and their microenvironment.

**What changes do you think could improve the professional lives of early-career scientists?**

Reducing pressure on postdocs to travel in order to gain experience. It can often be difficult for scientists with young families to continually uproot them when working short-term contracts early in their careers. This also applies for scientists no longer living with their children who still want to have an active part in their upbringing. This could be taken into account when considering job applications from candidates who have worked in few institutions. Some PIs might see this as a lack of ambition, but for early-career scientists it may be the only option to continue a career in science and still maintain a healthy relationship with their families outside of work.

**What's next for you?**

I will soon be starting a postdoc investigating the link between diabetes and colorectal cancer using metabolomics and *in vivo* models. In the future, I would like to apply for fellowships with the goal of running my own lab.

**Twitter handle**

@leggey86
